# The influential role of parents’ socioeconomic status and diet quality on their children’s dietary behavior: results from the LIPOKAP study among the Iranian population

**DOI:** 10.1186/s12887-023-04000-6

**Published:** 2023-04-21

**Authors:** Elham Karimi, Fahimeh Haghighatdoost, Noushin Mohammadifard, Farid Najafi, Hossein Farshidi, Tooba Kazemi, Kamal Solati, Farnaz Shahdadian, Arman Arab, Nizal Sarrafzadegan, Marjan Mansourian

**Affiliations:** 1 Department of Clinical Nutrition, School of Nutrition and Food Sciences, Isfahan University of Medical Sciences, Isfahan, Iran; 2 Research Development Center, Arash Women’s Hospital, Tehran University of Medical Sciences, Tehran, Iran; 3 Hypertension Research Center, Cardiovascular Research Institute, Isfahan University of Medical Sciences, Isfahan, Iran; 4 Isfahan Cardiovascular Research Center, Cardiovascular Research Institute, Isfahan University of Medical Sciences, Isfahan, Iran; 5 Research Center for Environmental Determinants of Health, Health Institute, Kermanshah University of Medical Sciences, Kermanshah, Iran; 6 Cardiovascular Research Center, Hormozgan University of Medical Sciences, Bandarabbas, Iran; 7 Cardiovascular Disease Research Center, Birjand University of Medical Sciences, Birjand, Iran; 8 Department of Psychiatry, Shahrekord University of Medical Science, Shahrekord, Iran; 9 Department of Community Nutrition, School of Nutrition and Food Sciences, Isfahan University of Medical Sciences, Isfahan, Iran; 10 School of Population and Public Health, Faculty of Medicine, University of British Columbia, Vancouver, Canada; 11 Department of Epidemiology and Biostatistics, Health School, Isfahan University of Medical Sciences, Isfahan, Iran; 12 Pediatric Cardiovascular Research Center, Cardiovascular Research Institute, Isfahan University of Medical Sciences, Isfahan, Iran

**Keywords:** Diet quality, Nutrition Knowledge, Socio-economic status, Children, Iran

## Abstract

**Purpose:**

There is a paucity of evidence regarding the influential factors on Iranian children’s diet quality. To assess this issue, we explore the relationship between parental diet quality, socioeconomic status (SES), and nutritional knowledge with their children’s diet quality using a cross-sectional sample of the Iranian population.

**Methods:**

In this study, paired parents along with one of their children (aged 6–18 years old) who lived with them were included. Dietary intake was assessed using a food frequency questionnaire (FFQ) and diet quality was determined using an Alternative Healthy Eating Index-2010 (AHEI). Nutritional knowledge and SES were also explored using validated questionnaires. Multiple linear regression analysis was used and beta (β) and corresponding 95% confidence interval (CI) were reported.

**Results:**

After controlling for potential confounders, the parents’ AHEI was significantly associated with their children’s diet quality (β = 12.34, 95% CI: 10.75, 13.93; P < 0.001). The nutritional knowledge of parents was significantly associated with children’s AHEI after controlling for potential confounders (β = 1.63, 95% CI: 0.14, 3.11; P = 0.032). Moreover, the parents’ SES was inversely associated with the AHEI of children (β=-3.76, 95% CI: -5.40, -2.11; P < 0.001); however, further adjustment for confounders attenuated this relationship (Model 3: β = 0.87, 95% CI: -0.76, 2.34; P = 0.269).

**Conclusions:**

We found that the children’s diet quality could be influenced by their parents’ SES, nutritional knowledge, and diet quality. Our findings suggest that improving the nutritional knowledge of parents not only may improve the healthy eating pattern of parents but also could influence their children’s diet quality.

## Introduction

A healthy diet during childhood and adolescence, in addition, to promoting optimal growth, may play a considerable role in preventing non-communicable diseases (NCDs) in later life [1, 2]. On the other hand, the epidemic of non-communicable diseases is considered a global health concern, especially in developing countries [3, 4]. As a developing country, Iran also has a high prevalence of obesity and related disorders in children and adolescents [5–7]. Findings showed that there are various influential factors related to children’s eating habits in early life [8]. Therefore, evaluating the diet quality of children and adolescents, and identifying these factors is of great importance in terms of public health priorities.

The formation of eating habits in children is influenced by various interactions in different fields [9, 10]. These include several levels, from the closest environment to which the child interacts, such as family, peers, and school, to broader and external environmental factors, including shared culture, history, customs, and economic systems [11, 12]. Among these, children and adolescents have the most interaction with their families as gatekeepers of children’s food choices, whose eating habits may be influenced by socioeconomic status (SES), nutritional knowledge, and food choices of their parents. In particular, low SES is related to poorer health and development for children (4). The unfavorable SES may adversely contribute to metabolic disorders such as type 2 diabetes and obesity [13]. Socio-economic inequalities are also related to lifestyle, including eating habits and physical activity, and consequently weight status [10, 14]. Also, parents’ nutritional knowledge seems to play a crucial role in the selection of food options by the children [15–17].

Parents’ perceptions of children’s habits have been extensively studied in high-income and developed countries. A study in the UK reported that factors such as food costs, time constraints, lack of knowledge, and conflict management over family food choices affect a child’s food choices [18]. Another study found that parents regarded food advertising, extended family, and peer influence as motives for their children’s food choices [19]. Australian mothers also found that food availability, nutrition strategies, eating modeling, and food opportunities determine what their children eat [20]. Nevertheless, the lack of studies on the factors affecting the eating habits of children and adolescents in developing and middle-income countries [11] such as Iran has undermined effective planning and interventions.

In order to develop healthy food options in Iranian children and adolescents, it is crucial to understand the complexity of the factors that affect the food choices of this age group. Hence, the present study aimed to examine the possible role of parents’ diet quality, nutritional knowledge, and SES concerning the diet quality of their children using a cross-sectional sample of the Iranian population.

## Materials and methods

### Study Design and participants

The current study is presented as part of a Knowledge And Practice Of dyslipidemia prevention, management, and control (LIPOKAP) survey which is described in detail elsewhere [21]. The present cross-sectional study was performed between February 2018 to July 2019. In the LIPOKAP study, 2,456 individuals aged > 18 years were selected from 5 counties throughout Iran including Birjand in the east, Bandarabas in the south, Kermanshah in the west, and Isfahan and Shahrekord in the center via stratified multi-stage random cluster sampling approach. In the first step, people were classified on the basis of population distribution in rural and urban areas. Then, clusters within each county were selected randomly from a list of health centers. Finally, subjects were selected randomly within each cluster utilizing their national identification numbers. Participants were excluded if they had any systemic or dyslipidemia-related disorders, chronic kidney disease, liver disease, cancers, and immune system disorders. Furthermore, one of their children or adolescents of 850 adult participants (aged 6–18 years old), who lived with them were included in the LIPOKAP study [21]. Thus, in the current study, we allocated 850 adults and their children. Due to a lack of data and under or over-reporting of dietary intake (24-h total energy intake less than 800 and over 4200 Kcal/ day), we excluded 38 paired parents and their children in our analysis. A written informed consent form was obtained from a parent and/or legal guardian of the study participants. This study was approved by the Ethics Committee of Isfahan University of Medical Sciences (registration number: IR.MUI.RC.1395.4.077).

### Demographic and socioeconomic characteristics

Sociodemographic information of the participants including age, gender, smoking, and SES was collected using a self-administered questionnaire. SES was assessed through the following questions: educational level and the job of the household’s head, income, number of family members, homeownership status, being the owner of a car, number of cars, and number of trips in the past year. The nutritional knowledge of parents was assessed by using a validated questionnaire that was described previously [21]. Also, physical activity was assessed by the International Physical Activity Questionnaire (IPAQ) and metabolic equivalent hours per week (MET-h / week) were calculated for children and adolescents [22].

### Dietary intake

The dietary intake of participants was assessed by a 110-item semi-quantitative food frequency questionnaire (SFFQ) and its validity and reliability were explained in detail in a previously published study [23]. This SFFQ contained information on the frequency of consumption and portion sizes of foods and dishes in the past year. The daily amount in grams for each food item was calculated. Individuals were asked regarding their consumption frequency of each food item with the following options: ‘seldom/never’, ‘1 per month’, ‘2–3 per month’, ‘1 per week’, ‘2–3 per week’, ‘4–6 per week’, ‘1 per day’, ‘2–3 per day’, ‘4–5 per day’, and ‘6 or more per day’. Seldom and never were considered to be “zero”. Dietary analysis regarding macro- / micro-nutrients was carried out via Nutritionist 4 software (First Databank, Hearst Corp, San Bruno, CA, USA), which was modified for Iranian foods.

### Alternative healthy eating index (AHEI)

To determine adherence to the healthy eating guidelines, we used AHEI-2010 [24]. AHEI consists of 11 components: whole grains, nuts and legumes, fruit, vegetables, long-chain omega-3 fatty acids fats [Docosahexaenoic acid (DHA) and Eicosapentaenoic acid (EPA)], polyunsaturated fatty acid (PUFA), trans-fatty acid (TFA), sugar-sweetened beverages (SSBs) and fruit juice, red and processed meats, alcohol consumption and sodium. In the present study, alcohol and sodium were not included in the calculation of AHEI. Alcohol is prohibited in Iran owing to religious issues. Moreover, we used a 110-item SFFQ which is not a valid approach to assess dietary intakes of sodium. Firstly, energy-adjusted values of each food item were calculated via the residual method [25]. Six components including whole grains, nuts and legumes, fruit, vegetables, long-chain omega-3 fatty acids (DHA and EPA), and PUFA scored 10 with the highest consumption and 1 with the lowest one. In contrast, three other components (TFA, SSBs and fruit juice, red and processed meats) scored 10 with the lowest consumption and 1 with the highest consumption. Finally, the whole AHEI-2010 was obtained by summing up the scores of nine components ranging from 9 to 90.

### Statistical analysis

Prior to data analysis, the normal distribution of continuous variables was examined via skewness statistic, histogram chart, and Kolmogorov-Smirnov test. Categorical and continuous variables were presented as number (percentage) and mean ± standard error (SE), respectively. The Chi-square test was implemented to examine the distribution of categorical variables across tertiles of parents’ AHEI, SES, and nutritional knowledge. Differences in continuous variables throughout the tertiles of parents’ AHEI, SES, and nutritional knowledge were assessed using a one-way analysis of variance (ANOVA). To assess the relationship between the parents’ AHEI, SES, and nutritional knowledge with children’s AHEI, linear regression analysis was performed and beta (β) with a corresponding 95% confidence interval (CI) was presented. First, we adjusted for the age (continuous) and sex of children, the age of parents (continuous), and SES (continuous), and then we made further adjustments for physical activity (continuous), smoking (yes/no), and 24-h total energy intake of children (continuous), AHEI (continuous), nutritional knowledge (continuous), and 24-h total energy intake of parents (continuous). Data analysis was performed using Statistical Package for Social Sciences (SPSS) version 26 (IBM Corp, Armonk, NY, USA). P-values < 0.05 were considered statistically significant.

## Results

General characteristics of children across the tertiles of parents’ AHEI are presented in Table 1. Overall, 822 children and adolescents were enrolled in our study. As can be seen, significant differences were found in terms of SES, total energy intake, and AHEI of parents across tertiles of parents’ AHEI (all P values < 0.05). In other words, children in the last tertile of parents’ AHEI have significantly lower SES scores and total energy intake.

**Table 1:** General characteristics of children across tertiles of Parents’ Alternative Healthy Eating Index

	Tertiles of parents’ AHEI			P-value
	**T1 (N = 275)**	**T2 (N = 278)**	**T3 (N = 269)**	
Age (Year)	11.93 ± 0.22	11.96 ± 0.24	11.71 ± 0.24	0.715
Sex (Female) n (%)	147 (53.5	139 (50.0	137 (50.9)	0.703
SES	12.23 ± 0.15	11.25 ± 0.17	9.45 ± 0.14	< 0.001
Physical activity (MET/minute day)	64.52 ± 5.25	73.03 ± 5.59	82.33 ± 5.68	0.075
Current smoker n (%)	3 (1.1)	5 (1.8)	9(3.3)	0.168
Parents AHEI	37.76 ± 0.30	49.27 ± 0.16	61.66 ± 0.30	< 0.001
Energy (kcal)	2490.68 ± 56.12	2363.55 ± 54.07	2251.84 ± 50.42	0.007

AHEI: Alternative Healthy Eating Index, SES: socioeconomic status

Data are presented as mean ± standard error or number (percent)

P < 0.05 was considered statistically significant

The P-value was obtained from the Chi-square test for categorical variables or a one-way analysis of variance (ANOVA) for continuous variables

The results of the children’s AHEI and its components across the tertiles of parents’ AHEI, SES, and nutritional knowledge are provided in Table 2. Children in the highest tertile of parents’ AHEI consumed higher amounts of fruit, vegetables, nuts, seeds and legumes, whole grains, PUFA, DHA, and EPA as well as lower amounts of red and processed meats, SSBs, and TFA compared to the lowest tertile (all P values < 0.05). In addition, children in the third tertile of parents’ SES, consumed lower amounts of fruit, vegetables, nuts, seeds, and legumes, whole grains, red and processed meats, and EPA and DHA compared to those in the first tertile (all P values < 0.05). Additionally, children of the first tertile of parents’ SES, had lower consumption for SSB, TFA, and PUFA compared to the last tertile (all P values < 0.05). In terms of parents’ nutritional knowledge, children in the highest tertile consumed higher amounts of fruit (P = 0.08) and vegetables (P = 0.006), and lower amounts of SSB (0.059) and TFA (0.054) compared to the lowest tertile.

**Table 2:** AHEI and its components of children across tertiles of parents’ Alternative Healthy Eating Index, Socioeconomic status, and nutritional knowledge

	Tertiles of parents’ AHEI			P-value
	**T1(N = 275)**	**T2 (N = 278)**	**T3 (N = 269)**	
Children AHEI	43.96 ± 0.56	49.76 ± 0.55	54.88 ± 0.48	< 0.001
Fruits (gr/ day)	130.25 ± 5.34	203.66 ± 6.38	288.36 ± 8.32	< 0.001
Vegetables (gr/ day)	214.91 ± 5.58	285.78 ± 7.72	389.88 ± 10.23	< 0.001
Nuts, seeds, and legumes (gr/ day)	56.45 ± 2.76	88.74 ± 3.52	112.78 ± 3.63	< 0.001
Whole grains (gr/ day)	60.06 ± 6.27	89.43 ± 6.72	97.63 ± 6.58	< 0.001
Red and processed meat (gr/ day)	52.11 ± 3.34	45.02 ± 2.26	31.70 ± 2.03	< 0.001
SSB (gr/ day)	197.28 ± 13.52	106.05 ± 9.34	55.43 ± 5.38	< 0.001
TFA (gr/ day)	2.78 ± 0.18	1.33 ± 0.12	0.54 ± 0.04	< 0.001
PUFA (gr/ day)	26.01 ± 0.63	26.80 ± 0.63	28.92 ± 0.62	0.004
EPA + DHA (gr/ day)	0.10 ± 0.01	0.12 ± 0.009	0.14 ± 0.009	0.013
	Tertiles of parents’ SES			**P-value**
	**T1 (N = 273)**	**T2 (N = 274)**	**T3 (N = 275)**	
Children AHEI	51.41 ± 0.60	49.42 ± 0.62	47.69 ± 0.55	< 0.001
Fruits (gr/ day)	224.95 ± 6.96	218.00 ± 8.32	172.22 ± 7.54	< 0.001
Vegetables (gr/ day)	304.27 ± 9.83	311.83 ± 8.84	266.48 ± 8.15	0.001
Nuts, seeds, and legumes (gr/ day)	94.84 ± 3.34	87.15 ± 3.79	73.49 ± 3.53	< 0.001
Whole grains (gr/ day)	104.04 ± 7.38	79.07 ± 6.17	62.71 ± 6.00	< 0.001
Red and processed meat (gr/ day)	59.29 ± 3.02	48.63 ± 2.86	21.64 ± 1.22	< 0.001
SSB (gr/ day)	90.58 ± 8.84	125.37 ± 11.66	149.15 ± 11.49	< 0.001
TFA (gr/ day)	0.97 ± 0.7	2.00 ± 0.16	1.77 ± 0.17	< 0.001
PUFA (gr/ day)	24.92 ± 0.54	27.19 ± 0.61	29.46 ± 0.71	< 0.001
EPA + DHA (gr/ day)	0.16 ± 0.01	0.13 ± 0.01	0.07 ± 0.005	< 0.001
	Tertiles of parents’ nutritional knowledge			**P-value**
	**T1 (N = 295)**	**T2 (N = 303)**	**T3 (N = 224)**	
Children AHEI	48.70 ± 0.58	48.77 ± 0.57	51.51 ± 0.63	0.002
Fruits (gr/ day)	194.86 ± 7.91	203.62 ± 7.34	219.74 ± 7.73	0.088
Vegetables (gr/ day)	288.25 ± 8.46	279.78 ± 8.65	320.54 ± 9.96	0.006
Nuts, seeds, and legumes (gr/ day)	83.49 ± 3.92	83.89 ± 3.31	89.00 ± 3.32	0.451
Whole grains (gr/ day)	76.37 ± 6.12	77.38 ± 6.35	95.23 ± 7.48	0.10
Red and processed meat (gr/ day)	39.03 ± 2.67	45.51 ± 2.88	46.02 ± 2.18	0.103
SSB (gr/ day)	119.82 ± 10.76	138.52 ± 10.48	101.19 ± 10.91	0.059
TFA (gr/ day)	1.65 ± 0.15	1.72 ± 0.14	1.29 ± 0.13	0.054
PUFA (gr/ day)	26.92 ± 0.65	27.73 ± 0.62	26.82 ± 0.61	0.532
EPA + DHA (gr/ day)	0.11 ± 0.01	0.12 ± 0.008	0.13 ± 0.01	0.765

AHEI: alternative healthy eating index, SES: socioeconomic status, SSB: sugar-sweetened beverages, TFA: trans-fatty acid; PUFA: polyunsaturated fatty acid, EPA: eicosatetraenoic acid, DHA: docosahexaenoic acid

Data are presented as mean ± SE

P < 0.05 was considered statistically significant

The P-value was obtained from a one-way analysis of variance (ANOVA).

The findings of linear regression of parents’ AHEI, SES, and nutritional knowledge with AHEI of children are shown in Table 3. The parents’ AHEI was significantly associated with their children’s diet quality (β = 10.92, 95% CI: 9.42, 12.41; P < 0.001) in the crude model. This association remained also significant after adjustment for age, sex, physical activity, smoking, and energy intake of children, and also the age of parents and SES (β = 12.34, 95% CI: 10.75, 13.93; P < 0.001). The nutritional knowledge of parents was significantly associated with children’s AHEI even after controlling for potential confounders (β = 1.63, 95% CI: 0.14, 3.11; P = 0.032). Moreover, the parents’ SES was inversely associated with AHEI of children (β=-3.76, 95% CI: -5.40, -2.11; P < 0.001); however, further adjustment for, AHEI, nutritional knowledge, and energy intake of parents, this relationship was not observed (Model 3: β = 0.87, 95% CI: -0.76, 2.34; P = 0.269).

**Table 3:** Beta (β) and 95% confidence interval for Alternative Healthy Eating Index of children according to tertiles of parents’ Alternative Healthy Eating Index, socioeconomic status, and nutritional knowledge

	Tertiles of parents’ AHEI			
	**T1**	**T2**	**T3**	**P trend**
Children’s AHEI Model 1 Model 2 Model 3	Ref Ref Ref	5.80 (4.32, 7.28) 5.86 (4.36, 7.36) 6.81 (5.35, 8.26)	10.92 (9.42, 12.41) 11.19 (9.57, 12.81) 12.34 (10.75, 13.93)	< 0.001 < 0.001 < 0.001
	Tertiles of parents’ SES			
	**T1**	**T2**	**T3**	**P trend**
Children’s AHEI Model 1 Model 2 Model 3	Ref Ref Ref	-1.98 (-3.63, -0.33) -2.15 (-3.81, -0.50) -0.02 (-1.45, 1.41)	-3.71 (-5.36, -2.06) -3.76 (-5.40, -2.11) -0.87 (-0.76, 2.34)	< 0.001 < 0.001 0.274
	Tertiles of parents’ nutritional knowledge			
	**T1**	**T2**	**T3**	**P trend**
Children’s AHEI Model 1 Model 2 Model 3	Ref Ref Ref	0.07 (-1.51, 1.65) 0.06 (-1.50, 1.64) 0.09 (-1.26, 1.45)	2.81 (1.09,4.52) 2.83 (1.12, 4.54) 1.63 (0.14, 3.11)	0.002 0.003 0.039

AHEI: Alternative Healthy Eating Index, SES: socioeconomic status

Data are presented as Beta (β) (95% confidence interval)

P < 0.05 was considered statistically significant

Model 1: Unadjusted

Model 2: Adjusted for age and sex of children, age of parents, and SES

Model 3: Model 2 + physical activity, smoking, and energy intake of children, AHEI, nutritional knowledge, and energy intake of parents

## Discussion

In this large population-based study conducted among the Iranian population, we found that the parents’ AHEI and nutritional knowledge are independent predictors of their children’s AHEI. Of note, the association between parents’ SES and their children’s AHEI was not independent of children’s physical activity, energy intake, smoking, and their parents’ AHEI, nutritional knowledge, and energy intake.

Previously, parents thought that their children’s food preferences are the main influential factor for their children’s food choices. This finding was built upon the previous investigation in a middle-income country [26] and also a systematic review among high-income countries [27]. Furthermore, taste and appearance as justification for food preference have been reported previously [18, 28]. Parents believed that children do not poses a clear-cut understanding of diet and healthy choices, which led to the eating of preferred foods only. However, earlier documents suggested that children recognize the concept of nutrition to categorize foods as unhealthy or healthy [29, 30].

Applying the nutritional knowledge of the parents and also their practice, which was measured by AHEI in the current study, were identified as two of the core influential factors on children’s AHEI in the current study. This finding was consistent with the previous reports among urban Sri Lankan preschool children. In this study, 110 parents of children aged 2–6 years were assessed regarding the most influential factors in their children’s food choices. The findings proposed that nutritional knowledge and the practice of caregivers are among the most influential factors [31]. A recent systematic review by Sirasa et al. concluded that family income, nutritional knowledge of family or caregivers, and household food availability were all associated with healthy food consumption in children. Likewise, children’s unhealthy food consumption was also linked with family or caregivers’ nutritional knowledge [32]. Furthermore, it has been reported that poor nutritional knowledge in parents contributed to unhealthy food choices in their children [27]. A logical explanation regarding this issue could be parenting education (i.e. explaining healthy foods to children) and limiting the unhealthy food choices in the household by removing these food items from the shopping list [26, 28]. This suggests that educating parents and caregivers about various aspects of child nutrition including how to nourish a child properly, the nutrient requirements of a child, and the importance of nutrition can provide a great opportunity for parents and caregivers to apply their knowledge in feeding their child [33, 34].

Moreover, we found that parents’ SES was associated with their children’s AHEI but disappeared after adjustment for AHEI, nutritional knowledge, and energy intake of parents. This finding emphasizes that parents’ SES is less important than their nutritional knowledge and AHEI and also was in contrast with previous reports. A qualitative study was conducted among thirty-eight low-income mothers in Argentina to examine the influence of parents’ SES on their children’s eating practices. The results indicated that lower SES scores influence children’s feeding practice which was inconsistent with our findings [26]. However, due to the low sample size of the recent study and also the cultural differences between Iran and Argentina, the comparison is difficult. Moreover, a previous report from the British birth cohort ALSPAC revealed that children with junk dietary patterns were more probably to live in lower-income families with less educated mothers [35]. Among the subsets of SES, it has been reported that family income is the most influential factor in the food choices of children [31]. Furthermore, nearly 30% of children believed that financial problems are among the most important obstacle regarding healthy and adequate amounts of food purchasing [26, 27]. The household food preparation facilities are another factor regarding children’s food choices which is related to family income. There are some explanations for these controversial findings. First, there are some cultural differences between the Iranian population and previous studies which makes it difficult to compare the results. Moreover, higher family income which is one of the important subsets of SES can cause a family to eat more in a restaurant than at home. More frequent consumption of food in restaurants increases the consumption of fast food and reduces the quality of the diet, which can justify the results of the present study.

Parents and especially mothers play a crucial role in modeling the eating patterns of children. Usually, mothers spend more time with their children during the meal than fathers do, and most of the time, they are known as the family food supplier [36, 37]. In contrast, the influential role of mothers is diminished among adolescents, indicating that the nutrition-controlling role of parents is lost once their children become teenagers. A study by Vilela et al. among Portuguese families indicated that mothers with higher education are a significant predictor of lower consumption of red and processed meat among children, but not adolescents [37]. However, the distribution of children and adolescents was not similar in our study which precludes us to do subgroup analysis on the basis of children/adolescents. Hence, it is strongly suggested that future studies examine this issue separately for different age groups.

In addition to the studied parameters, other factors may influence the dietary intake of children and adolescents. Household food availability was shown to be positively associated with children’s micronutrient intake and healthy food consumption [32]. Moreover, the engagement of parents or caregivers in a particular type of food production activity increased the likelihood of a parent or caregiver feeding his/her young child more of that food. However, this influential factor may diminish as children get older [32]. Moreover, parental education, family rules, and parental encouragement are also suggested to affect children’s dietary intake [38, 39]. Likewise, the association between food exposure in the school and home environment with healthy food consumption among children was proposed [40, 41].

### Strength and limitations

To the best of our knowledge, the current study is the first population-based investigation among Iranians to assess the possible parental predictors of children’s diet quality. Also, we used a large nationally representative sample of the Iranian population by sampling from 5 different geographical areas of Iran. This study has also several limitations. First, the present study was a cross-sectional sample of the Iranian population; hence, it precludes us from examining the causality. Second, there are other factors including anthropometric measures which were not measured in the current study. Third, although we adjusted for several demographic and nutritional factors, we cannot exclude the possible effect of residual confounding on our results. Fourth, dietary intakes of children and their parents were examined via the same SFFQ and therefore may introduce another source of bias in estimating precise dietary intakes. Fifth, no information regarding food availability in schools was examined and therefore future studies should take this point into account.

## Conclusion

In conclusion, we found that the children’s diet quality could be influenced by their parents’ SES, nutritional knowledge, and diet quality. Our findings suggest that improving the nutritional knowledge of parents not only may improve the healthy eating pattern of parents but also could influence their children’s diet quality. Additional longitudinal and interventional studies are needed to confirm these findings and draw a firm link in this regard.

## Data Availability

The data that support the findings of this study are available from the corresponding author upon reasonable request.
